# Associations of Dietary Patterns and Nutrients with Glycated Hemoglobin in Participants with and without Type 1 Diabetes

**DOI:** 10.3390/nu13031035

**Published:** 2021-03-23

**Authors:** Arpita Basu, Amy C. Alman, Janet K. Snell-Bergeon

**Affiliations:** 1 Department of Kinesiology and Nutrition Sciences, School of Integrated Health Sciences, University of Nevada, Las Vegas, NV 89154, USA; 2 College of Public Health, University of South Florida, Tampa, FL 33612, USA; aalman@usf.edu; 3 Barbara Davis Center for Childhood Diabetes, Anschutz Medical Campus, University of Colorado, Aurora, CO 80045, USA; janet.snell-bergeon@cuanschutz.edu

**Keywords:** dietary patterns, baked desserts, type 1 diabetes, glycated hemoglobin

## Abstract

Background: Diet has been associated with poor glycemic control in diabetes. Few studies have examined this association in people with type 1 diabetes (T1D), who are at a higher risk for cardiovascular disease than people without diabetes. Methods: We report data from cross-sectional and longitudinal analyses from a coronary artery calcification in type 1 diabetes (CACTI) study (*n* = 1257; T1D: *n* = 568; non-diabetic controls: *n* = 689) collected between the years 2000 and 2002. Participants completed a validated food frequency questionnaire, a physical examination, and biochemical analyses. Dietary patterns based on variations in food group intake were created with principal components analysis. Linear regression was used to examine the associations of dietary patterns, macronutrients, and food groups with HbA1c in a model adjusted for relevant covariates and stratified by diabetes status. Results: Three dietary patterns were identified: “fruits, veggies, meats, cereal”, “baked desserts” and “convenience foods and alcohol” patterns. At baseline, a higher intake of the “baked dessert” pattern was significantly associated with higher HbA1c in T1D at baseline as well at year 6 of the study when adjusted for age, sex, BMI, total calories, and diabetes duration. No such associations were observed in the case of non-diabetic controls. Dietary saturated fats and animal fats were also positively associated with HbA1c in adults with T1D at baseline and/or at year 6. Conclusions: The habitual intake of a dietary pattern that is characterized by an increased intake of added sugar and saturated fats, such as in baked desserts, may increase risks of poor glycemic control in T1D.

## 1. Introduction

The prevalence of diabetes mellitus has been increasing at an alarming rate in the US adult population [1], including type 1 diabetes (T1D), which carries a higher burden of cardiovascular disease (CVD) than is found in the general population [2]. T1D is an autoimmune condition characterized by pancreatic beta cell destruction and absolute insulin deficiency, and dietary measures remain a key for glycemic control and achieving glycated hemoglobin HbA1c ≤ 7.0% in the primary management of this disease [3]. The American Diabetes Association has increasingly emphasized the need for “healthful eating patterns” that contain a variety of nutrient-dense foods for optimal glycemic control, as well as in the management of obesity and dyslipidemia, which contribute to excess CVD risks in diabetes [4]. Despite several reported clinical trials on the optimal diet composition for the management of hyperglycemia in T1D, the results remain inconclusive in most cases. Dietary carbohydrates as a direct contributor to the glycemic load have been historically targeted as a goal of diabetes management, and thus low-carbohydrate diets have gained much attention [5]. However, by restricting a single nutrient- and carbohydrate-containing food group, such as whole grains, fruits and dairy may also be omitted, thereby leading to the exclusion of many micronutrients and dietary bioactive compounds shown to improve hyperglycemia [6,7]. This necessitates the examination of dietary patterns over nutrients.

While the role of dietary patterns has been associated with glycemic control and vascular complications of T1D in children and adolescents [8,9], only a few studies have reported the association of dietary patterns with glycemic control in adults. In a cross-sectional study in Chinese adults with T1D (*n* = 99), a higher intake of a dietary pattern containing high-fat cakes, beans and picked vegetables was positively associated with HbA1c and LDL-cholesterol when compared to those consuming a diet high in rice and eggs [10]. In a seven-year prospective cohort study in European adults with T1D (*n* = 1659), a lower intake of vegetable, protein and fiber was inversely associated with HbA1c; this study did not report dietary patterns and micronutrient intakes [11]. In another prospective study with a five-year follow-up in T1D adults residing in Canada and the US (*n* = 532) undergoing intensive treatment from the Diabetes Control and Complications Trial (DCCT), inverse associations between HbA1c and dietary carbohydrate, and positive associations with total fat, saturated fatty acids and mono-unsaturated fatty acids intake, were shown [12]. Again, dietary patterns were not reported in the DCCT report. Studies assessing dietary patterns and how they relate to glycemic control have been reported in type 2 diabetes: western-style dietary pattern and diets containing sweets and dairy were associated with higher HbA1c in a cross-sectional study in Mexico (*n* = 4838) [13]; dietary patterns characterized by “refined grains and meat” and “oil and salt” were positively associated with insulin resistance in a case–control study in China (*n* = 836) [14]; again, a western-style dietary pattern was associated with hyperglycemia in a cross-sectional study in Iran (*n* = 400) [15].

Habitual dietary intakes vary widely among populations and by geographical regions. Thus, we sought to examine how dietary patterns are associated with glycemic control in a US population with and without type 1 diabetes participating in the coronary artery calcification in type 1 diabetes (CACTI) study. We aim to contribute to the dearth of literature on the role of dietary patterns and macronutrients with glycemic control in adults with and without T1D. We have previously reported an inverse association between dietary fiber intake and HbA1c in adults with and without T1D [16]. We extended this dietary investigation and sought to examine how dietary patterns, nutrients and food groups were associated with HbA1c in the same cohort.

## 2. Materials and Methods

Study participants: In this report we present data collected at baseline and at year 6 of the CACTI study as described previously [17]. All study participants provided informed consent and the study protocol was approved by the Colorado Multiple Institutional Review Board (ethics code 97-661). The study was registered at clinicaltrials.gov (NCT00005754).

Dietary intake: The dietary intakes of foods and nutrients were determined using a validated self-administered food-frequency questionnaire (Harvard, 1988) [18]. The procedures have been previously published in detail [17].

Cardiovascular risk factors: Baseline examinations were conducted between March 2000 and April 2002. Anthropometric measurements involved height, weight and waist circumference, and body mass index. Systolic blood pressure (SBP) and fifth-phase diastolic blood pressure (DBP) were measured during the rest state and an average of three measurements were reported. Participants also completed standardized questionnaires on medical history, current medication, insulin doses, physical activity (modifiable activity questionnaire) [19], alcohol and tobacco use and family medical history. Blood samples were collected in the 12 h fasting state for biochemical analyses (blood LDL- and HDL-cholesterol, triacylglycerol and HbA1c).

Statistical analysis: Baseline characteristics between men and women with type 1 diabetes and without diabetes were examined using a Student’s *t* test. A *χ*^2^ test for goodness of fit was used to determine if categorical risk factors differed between participants with and without diabetes. A Wilcoxon rank sum test was used to compare differences in continuous variables with skewed distributions. Factor analysis was used to formulate dietary patterns, and foods were considered to form part of the dietary patterns with a load factor greater than 0.4. The varimax rotation was made to generate robust dietary patterns for interpretation. We only considered factors with the three largest eigenvalues for further analyses. Linear regression analysis was used to examine the associations of dietary patterns and macronutrients with baseline HbA1c, as well as with HbA1c at the year 6 follow-up. Models were adjusted for relevant covariates as follows: model 1 (age, sex, BMI, and diabetes duration for T1D), model 2 (model 1 + total calories, dietary carbohydrates, fats, proteins, and fiber) and model 3 (model 1 + total calories and serum lipids). These models were stratified by diabetes as we observed significant interaction effects. In addition, longitudinal analyses were adjusted for baseline HbA1c, year 6 BMI and the duration of follow-up. We also examined the associations of food groups with HbA1c at baseline and at the year 6 follow-up in a model adjusted for age, sex, BMI, total calories, and diabetes status; the year 6 model was further adjusted for baseline HbA1c, year 6 BMI and duration of follow-up.

## 3. Results

### 3.1. Baseline and Year 6 Features

Table 1 shows the baseline characteristics of the cohort by diabetes status. Among the participants with T1D, we observed significantly younger age, a greater number of females, higher systolic but lower diastolic blood pressure, and better lipid profiles (lower LDL-C and triglycerides and higher HDL-C) when compared with non-diabetic controls. As expected, those with T1D had significantly higher HbA1c than the controls. While total calorie intake did not differ between groups, adults with T1D consumed significantly lower percentage of total calories as carbohydrates, but a higher percentage of calories as proteins, dietary fats, saturated fats as well as animal fats when compared to non-diabetic controls. No significant differences in BMI and physical activity levels were noted between groups (Table 1). At year 6, among adults with T1D, the mean ± SD for HbA1c was 8.1 ± 1.6%, BMI was 26.8 ± 4.5 kg/m^2^, SBP was 121 ± 16 mm Hg, DBP was 78 ± 12 mm Hg, LDL-C was 108 ± 32 mg/dL, HDL-C was 52 ± 10 mg/dL, and triacylglycerol was 102 ± 52 mg/dL. These values were not significantly different from baseline.

### 3.2. Associations of Dietary Patterns and Nutrients with Glycemia at Cross-Sectional and Longitudinal Time Points

Table 2 depicts the multivariate model examining the associations of dietary patterns and nutrients with HbA1c at baseline and at year 6 of the study in models adjusted for age, sex, BMI, total calories, and nutrients, as well as blood lipids, stratified by diabetes. Among the dietary patterns identified in the cohort, the “fruit, veggie, cereal and meat” pattern and the “convenience food and alcohol” pattern were not associated with HbA1c at any time point in participants with and without T1D. On the other hand, the “baked desserts” pattern was positively associated with baseline HbA1c in the model adjusted for age, sex, and diabetes duration, as well as in the model further adjusted for total calories and blood lipids in the T1D group. This pattern tended to be positively associated when the model was further adjusted for the macronutrients. Similarly, the “baked desserts” pattern was positively associated with year 6 HbA1c in all models and revealed a borderline significant association in the model further adjusted for total calories and blood lipids in the T1D group. No such associations were noted in non-diabetic controls. Among the macronutrients, dietary carbohydrates and proteins, as a percentage of total calories, revealed a significant positive association with year 6 HbA1c in the T1D group in models adjusted for age, sex, BMI, diabetes duration, total calories, and blood lipids, but not in the model further adjusted for other macronutrients and fiber. No such associations were noted in non-diabetic controls. Additionally, dietary carbohydrates and proteins revealed no associations with baseline HbA1c in any model. While total dietary fats revealed no association with HbA1c at any time point in the T1D group, saturated fats, as a percentage of total calories, revealed a significant positive association with baseline, as well as year 6 HbA1c only in the model adjusted for age, sex, BMI, and diabetes duration in the T1D group. Finally, animal fats also showed a significant positive association with baseline HbA1c in models adjusted for the aforementioned factors, as well as total calories and macronutrients. Vegetable fats revealed no significant associations with HbA1c at any time point in either group.

### 3.3. Associations of Food Groups with Glycemia at Cross-Sectional and Longitudinal Time Points

Table 3 depicts the multivariate model examining the associations of dietary food groups with HbA1c at baseline and at year 6 of the study in a model adjusted for age, sex, BMI, total calories, and diabetes status. Broadly, no significant associations were noted in the fruit and vegetable groups, except for an inverse association of dark green vegetables with baseline HbA1c and of tomatoes with year 6 HbA1c. No associations were noted for dairy, legumes, nuts, cereals, and protein groups. Interestingly, in the beverage group, low-calorie/no-calorie beverages were positively associated with HbA1c at both time points. As constituents of the “baked desserts” pattern, commercial and home-made cookies, candies, cakes, and pies as a food group also revealed a significant positive association with HbA1c at both time points.

## 4. Discussion

We report the significant positive association of a dietary pattern consisting mainly of baked desserts, such as candies, pies, cookies, and cakes, with HbA1c at baseline and at year 6 of our longitudinal study in adults with T1D. This positive association remained significant in models adjusted for age, sex, BMI, diabetes duration, total calories, and/or blood lipids (serum cholesterol and triglycerides). To our knowledge, no previous study has reported such a consistent association between the habitual intake of baked goods and glycemic control in T1D adults. Among the commonly consumed food groups, as revealed in a meta-analysis of prospective studies (*n* = 88), red and processed meats and sugar-sweetened beverages (SSB) were associated with increased risks of type 2 diabetes. The study examined this association among 12 food groups but did not address baked desserts or those containing added fats and sugars, except SSB [20]. A baked desserts diet pattern is typically high in refined grains, added sugars, especially corn syrup, fat, and sodium, and low in fibers that have been associated with increased risks of diabetes [21,22]. Baked desserts are also high in hydrogenated fats, which is an important source of trans fats shown to increase diabetes and CVD risks [23,24,25,26]. On the other hand, we observed no association of a healthier dietary pattern characterized by fruits, vegetables, cereals and meats, or a diet consisting of convenience foods and alcohol, with HbA1c in these adults, quite likely due to the foods in these diet patterns not significantly adding to or lowering the glycemic burden. These data show that in T1D adults, impaired glycemic control may be predicted by concentrated sources of sugars and fats, such as those in baked desserts, even if consuming lower dietary total carbohydrates than those without diabetes and following adjustment for total calories.

We also observed a positive association of saturated fats and animal fats with HbA1c at baseline and/or at year 6 of the study in adults with T1D. Reports from some prospective studies in T1D adults, such as the EURODIAB Prospective Complication study with a seven-year follow-up (*n* = 1102) [27], as well as the DCCT trial with a five-year follow-up, reveal a habitual dietary consumption that is high in total and saturated fats, but low in carbohydrates and fiber. In addition, the DCCT trial revealed this atherogenic diet pattern to be positively associated with HbA1c in adults undergoing intensive treatment to achieve optimal glycemic control [12]. Similar trends in the habitual consumption of a high-fat diet have also been observed in children with T1D [28]. This high-fat diet pattern may be the result of lowering dietary sources of carbohydrates that directly add to the glycemic load, and increasing animal-based food groups high in protein and saturated fats that often add more palatability and satiety to the diet. Consequently, we also observed dietary protein intake to be positively associated with HbA1c at year 6 of the study in adjusted models. The higher HbA1c concentrations in association with higher saturated fat intakes may also be explained by the detrimental effects of saturated fats on insulin signaling. High-fat meals impair insulin signaling, which results in a transient increase in insulin resistance in mechanistic studies [29]. A study by Rosenfalck et al. suggested that a low-fat diet reduces basal free fatty acid concentrations and improves peripheral insulin sensitivity in a clinical study of T1D adults [30]. Based on these observations, the dietary management of T1D must aim to reduce saturated fat and animal fat intake to improve glycemic control and prevent CVD complications.

Our observations on the inverse associations of individual fruits and vegetables, such as tomatoes and dark green leafy vegetables, with HbA1c conform to several observational studies on the protective associations of fruits and/or vegetables against type 2 diabetes [31,32,33]. These colorful fruits and vegetables are typically rich in carotenoids, and serum carotenoids have been inversely associated with poor glycemic control in adults [34,35]. In animal models of diabetes, the oral administration of lycopene ameliorated diabetes-related and oxidative stress markers [36,37]. However, such reports are scarce in adults with T1D, a gap which is addressed by our study. These findings warrant further attention in clinical studies involving the supplementation of fruits and vegetables or their bioactive compounds in diabetes management. We also observed a consistent positive association of the habitual intake of low-calorie beverages with HbA1c at baseline, as well as at year 6 of the study in T1D adults. As a substitute for plain water and sugar-sweetened beverages, low-calorie and no-calorie beverage consumption is a palatable and perceivably healthy option. However, the evidence supporting the risk to benefit ratio of regularly consuming these beverages is quite mixed [38], and data have shown some low-calorie sweeteners can increase body weight comparable to sucrose in adults with overweight or obesity [39], as well as higher energy intake in youths with type 2 diabetes, making them prone to obesity and poor glycemic control [40]. Thus, caution must be exercised in their regular consumption in T1D management.

Our study has some limitations. First, dietary data at baseline from the FFQ relied on a retrospective self-report which may be prone to recall bias. Additionally, our current analysis did not involve dietary exposures at year 6 of the study. Second, these observed associations cannot be generalized to adults without T1D and with multiple comorbidities. Third, our baseline for data collection on dietary and nutrient intakes was more than a decade earlier, and this may cause some discrepancies with the dietary pattens of adults in the current timeframe. However, these dietary patterns are similar to the US dietary intakes evaluated over 17 years, revealing a persistent high intake of saturated fats and low-quality carbohydrates [41]. Additionally, due to the smaller sample size at year 6 compared to the baseline, due to follow-up loss of participants, the results could have been driven towards null findings. Fourth, we did not adjust for any new glucose control therapies during the six-year period that may have affected some of the null findings at year 6. Finally, we did not measure food-specific biomarkers, such as blood levels of trans fatty acids or polyphenols, to further validate the self-reported FFQ exposure data.

In conclusion, our findings identify a dietary pattern containing increasing amounts of baked desserts to be associated with increasing HbA1c levels at baseline, as well with HbA1c after six years of follow-up in adults with T1D. Baseline dietary saturated and animal fats also revealed similar associations. Among the food groups, the positive association of low-/no-calorie beverages adds to the lack of data on their cardiometabolic effects in diabetes in adults. These data provide evidence for limiting baked desserts, saturated and animal fats, as well as low-/no-calorie sweeteners, and increasing the consumption of colorful fruits and vegetables, in the medical nutrition therapy for glycemic control in T1D management.

## Figures and Tables

**Table 1:** Baseline characteristics of participants.

	Type 1 Diabetes		Non-Diabetic Controls		
	*n* = 568		*n* = 689		
Variables	Mean or *n*	SD or % ^§^	Mean or *n*	SD or % ^§^	*p* Value
Age (years)	37	9	39	9	**<0.0001**
Sex (female; *n*)	316	56	346	50	**0.031**
Diabetes duration (years)	23.5	8.9	--	--	--
HbA1c (%)	7.9	1.2	5.5	0.4	**<0.0001**
HbA1c (met < 7% goal)	113	20	N/A	N/A	N/A
BMI (kg/m^2^)	26.2	4.3	26.2	5.0	0.929
SBP (mmHg)	117	14	114	12	**<0.0001**
DBP (mmHg)	77	9	79	8	**0.001**
LDL-C (mg/dL)	101	29	115	33	**<0.0001**
HDL-C (mg/dL)	56	16	50	14	**<0.0001**
Triacylglycerol (mg/dL)	93	54	132	103	**<0.0001**
Dietary total calories (kcal/day)	1766	613	1822	619	0.111
Dietary carbohydrates (% kcal/day)	45	9	48	9	**<0.0001**
Dietary fats (% kcal/day)	35	7	33	7	**<0.0001**
Dietary saturated fats (% kcal/day)	12.7	3.1	11.7	2.8	**<0.0001**
Dietary proteins (% kcal/day)	19.7	3.6	18.5	3.9	**0.0007**
Dietary fiber (g/day)	16.7	7.8	16.7	8.1	0.874
Dietary animal fats (g/day)	40.7	20.1	38.1	18.6	**0.0188**
Dietary vegetable fats (g/day)	28.5	13.8	28.2	14.1	0.683
Fruit, veggie, cereal, and meat pattern	0.06	1.35	−0.06	0.56	0.07
Baked desserts pattern	0.01	1.18	−0.006	0.85	0.77
Convenience foods and alcohol pattern	−0.04	0.86	0.03	1.11	0.22
Physical activity (KJ/week)	6268	1121	6609	1098	0.21

^§^ Column percentage. *p* < 0.05 in bold font. N/A, not applicable.

**Table 2:** Associations of dietary patterns and macronutrients with HbA1c in adults with and without type 1 diabetes (T1D).

Dietary Patterns/Nutrients (Baseline)	HbA1c (Baseline)				HbA1c (Year 6)			
	T1D (*n* = 568)		Non-Diabetic Control (*n* = 689)		T1D (*n* = 452)		Non-Diabetic Control (*n* = 538)	
	β ± SE	*p*	β ± SE	*p*	β ± SE	*p*	β ± SE	*p*
Fruit, veggie, cereal, meat pattern								
Model 1 ^a^	0.06 ± 0.04	0.15	0.003 ± 0.03	0.91	0.03 ± 0.04	0.47	−0.005 ± 0.05	0.92
Model 2 ^b^	0.07 ± 0.04	0.10	0.016 ± 0.03	0.65	0.05 ± 0.04	0.19	−0.003 ± 0.05	0.94
Model 3 ^c^	0.03 ± 0.04	0.35	0.002 ± 0.03	0.94	0.02 ± 0.03	0.51	0.0002 ± 0.05	0.99
Baked desserts pattern								
Model 1 ^a^	0.11 ± 0.05	**0.02**	0.004 ± 0.03	0.96	0.12 ± 0.06	**0.03**	0.02 ± 0.02	0.32
Model 2 ^b^	0.08 ± 0.05	0.07	0.002 ± 0.03	0.98	0.11 ± 0.06	**0.04**	0.022 ± 0.02	0.37
Model 3 ^c^	0.11 ± 0.04	**0.02**	0.002 ± 0.02	0.89	0.11 ± 0.06	0.05	0.021 ± 0.02	0.41
Convenience foods and alcohol pattern								
Model 1 ^a^	0.12 ± 0.07	0.09	0.002 ± 0.05	0.65	0.06 ± 0.06	0.35	−0.007 ± 0.02	0.73
Model 2 ^b^	0.13 ± 0.07	0.06	0.001 ± 0.02	0.47	0.08 ± 0.06	0.18	−0.008 ± 0.02	0.75
Model 3 ^c^	0.06 ± 0.06	0.34	0.008 ± 0.02	0.64	0.05 ± 0.06	0.41	−0.011 ± 0.02	0.63
Carbohydrates (% kcal)								
Model 1 ^a^	0.006 ± 0.005	0.28	0.002 ± 0.001	0.18	0.013 ± 0.005	**0.02**	0.001 ± 0.002	0.46
Model 2 ^b^	0.017 ± 0.014	0.22	0.002 ± 0.003	0.56	0.002 ± 0.014	0.88	0.005 ± 0.003	0.27
Model 3 ^c^	0.007 ± 0.005	0.21	0.002 ± 0.001	0.23	0.014 ± 0.005	**0.02**	0.001 ± 0.002	0.47
Proteins (%kcal)								
Model 1 ^a^	0.01 ± 0.02	0.47	0.003 ± 0.004	0.40	0.03 ± 0.01	**0.02**	0.009 ± 0.005	0.86
Model 2 ^b^	0.03 ± 0.02	0.15	0.006 ± 0.005	0.23	0.03 ± 0.02	0.08	0.007 ± 0.004	0.66
Model 3 ^c^	0.02 ± 0.01	0.14	0.003 ± 0.002	0.42	0.03 ± 0.01	**0.008**	0.007 ± 0.003	0.86
Total Fats (% kcal)								
Model 1 ^a^	0.010 ± 0.007	0.17	0.003 ± 0.002	0.07	0.013 ± 0.007	0.08	0.006 ± 0.003	0.06
Model 2 ^b^	0.019 ± 0.015	0.19	0.009 ± 0.004	0.82	0.011 ± 0.15	0.43	0.013 ± 0.005	**0.02**
Model 3 ^c^	0.008 ± 0.007	0.21	0.003 ± 0.002	0.11	0.013 ± 0.007	0.07	0.006 ± 0.003	0.06
Saturated fats (% kcal)								
Model 1 ^a^	0.015 ± 0.002	**0.01**	0.001 ± 0.002	0.12	0.018 ± 0.007	**0.003**	0.002 ± 0.001	0.06
Model 2 ^b^	0.012 ± 0.015	0.09	0.009 ± 0.004	0.82	0.015 ± 0.04	0.22	0.014 ± 0.003	**0.02**
Model 3 ^c^	0.008 ± 0.003	0.09	0.002 ± 0.002	0.11	0.013 ± 0.007	0.13	0.010 ± 0.003	0.05
Animal fats								
Model 1 ^a^	0.008 ± 0.002	**0.002**	0.002 ± 0.002	0.11	0.004 ± 0.002	0.11	0.007 ± 0.02	0.88
Model 2 ^b^	0.009 ± 0.003	**0.006**	0.001 ± 0.001	0.17	0.006 ± 0.007	0.24	0.001 ± 0.01	0.92
Model 3 ^c^	0.006 ± 0.003	0.07	0.002 ± 0.001	0.06	0.004 ± 0.003	0.27	0.011 ± 0.01	0.44
Vegetable fats								
Model 1 ^a^	0.005 ± 0.003	0.15	0.003 ± 0.001	0.61	0.004 ± 0.003	0.19	0.003 ± 0.002	0.74
Model 2 ^b^	0.008 ± 0.003	0.84	0.002 ± 0.001	0.64	0.007 ± 0.006	0.26	0.008 ± 0.001	0.99
Model 3 ^c^	0.004 ± 0.003	0.91	0.003 ± 0.001	0.65	0.005 ± 0.004	0.44	0.003 ± 0.002	0.11

^a^ Model adjusted for age, sex, BMI, and diabetes duration for T1D; age, sex and BMI for non-diabetic control; year 6 model also adjusted for baseline HbA1c, year 6 BMI and duration of follow-up. ^b^ Model 1 + total calories, dietary carbohydrates, fats, proteins and fiber (excluding relevant covariates when these are independent variables in the model). ^c^ Model 1 + total calories, serum total cholesterol and triglycerides T1D: type 1 diabetes. *p* < 0.05 are in bold font.

**Table 3:** Associations of food groups with HbA1c in adults with and without T1D.

Food Groups (Baseline)	HbA1c (Baseline)		HbA1c (Year 6)	
	β ± SE	*p*	β ± SE	*p*
Whole fruits	0.001 ± 0.022	0.96	0.02 ± 0.01	0.19
Fruit juice	0.06 ± 0.04	0.14	0.06 ± 0.04	0.98
Tomatoes	−0.04 ± 0.07	0.61	−0.12 ± 0.04	**0.009**
Dark green vegetables	−0.04 ± 0.02	**0.04**	−0.02 ± 0.02	0.43
Starchy vegetables	−0.07 ± 0.06	0.31	−0.05 ± 0.04	0.19
Orange/red vegetables	0.05 ± 0.06	0.52	0.07 ± 0.03	0.85
Other vegetables	0.03 ± 0.04	0.51	0.05 ± 0.02	0.07
Low fat dairy	0.08 ± 0.06	0.17	−0.02 ± 0.04	0.64
Other dairy	0.03 ± 0.01	0.09	−0.03 ± 0.02	0.75
Legumes	−0.02 ± 0.06	0.67	0.11 ± 0.06	0.86
Nuts	0.10 ± 0.05	0.08	0.03 ± 0.05	0.40
Eggs	0.11 ± 0.05	0.99	0.03 ± 0.04	0.54
Red meat	0.05 ± 0.03	0.10	0.06 ± 0.03	0.66
Processed meat	0.03 ± 0.04	0.37	0.02 ± 0.01	0.27
Fish, low cholesterol	0.09 ± 0.05	0.79	0.05 ± 0.04	0.33
Fish, high cholesterol	0.11 ± 0.05	0.15	0.13 ± 0.05	0.15
Chicken/Turkey	0.03 ± 0.04	0.14	0.05 ± 0.04	0.81
Pizza	0.12 ± 0.05	0.97	0.11 ± 0.05	0.86
Salty snacks	0.02 ± 0.03	0.18	0.04 ± 0.03	0.17
Low-calorie/no-calorie beverages	0.11 ± 0.03	**0.002**	0.41 ± 0.06	**<0.001**
SSB	0.02 ± 0.01	0.47	0.07 ± 0.02	0.67
Tea	0.02 ± 0.03	0.51	0.11 ± 0.04	0.06
Coffee	−0.02 ± 0.02	0.33	−0.03 ± 0.04	0.47
Wine	−0.04 ± 0.04	0.35	0.05 ± 0.04	0.92
Beer	−0.06 ± 0.05	0.47	0.19 ± 0.05	0.79
Liquor	0.17 ± 0.10	0.08	0.11 ± 0.05	0.52
Candy, cookies, pies, cakes, rolls (homemade and commercial)	0.05 ± 0.01	**0.001**	0.03 ± 0.01	**0.04**
Cold breakfast cereal	0.09 ± 0.05	0.32	0.10 ± 0.06	0.06
Cooked breakfast cereal	0.04 ± 0.03	0.42	0.04 ± 0.03	0.21
Refined grains	0.05 ± 0.03	0.15	0.11 ± 0.03	0.54
Whole grains	0.02 ± 0.01	0.78	−0.06 ± 0.03	**0.03**

Adjusted for age, sex, BMI, total calories and diabetes status; year 6 model also adjusted for baseline HbA1c, year 6 BMI and duration of follow-up; SSB: sugar-sweetened beverage; T1D: type 1 diabetes. *p* < 0.05 are in bold font.

## Data Availability

The datasets analyzed in the current study are not publicly available due to ethical reasons and because our participants only gave their consent for the use of their data by the original team of investigators.
